# Retraction: Sorafenib combined with tarexib for first-line treatment of unresectable hepatocellular carcinoma and its predictive role and correlation with PD-L1 CTCs

**DOI:** 10.3389/fonc.2026.2021942

**Published:** 2026-10-27

**Authors:** Frontiers Editorial Office

The journal retracts the 2 December 2024 article cited above.

During post-publication review, it was noted that the article contains multiple errors in drug nomenclature. For example, Tislelizumab was incorrectly stated as Tarexib, Tirelizumab and Toripalimab. The editorial office contacted the corresponding author on multiple occasions requesting the raw data underlying these findings. The authors did not respond. As the validity of the article could not be verified, the article has been retracted.

This retraction was approved by the Chief Editors of Frontiers in Oncology and the Chief Executive Editor of Frontiers.

